# 4-{(*E*)-[2-(Pyridin-3-ylcarbon­yl)hydrazinyl­idene]meth­yl}phenyl acetate

**DOI:** 10.1107/S1600536813025075

**Published:** 2013-09-18

**Authors:** Riya Datta, V. Ramya, M. Sithambaresan, M. R. Prathapachandra Kurup

**Affiliations:** a Department of Chemistry, Christ University, Hosur Road, Bangalore 560 029, India; b Department of Chemistry, Faculty of Science, Eastern University, Sri Lanka, Chenkalady, Sri Lanka; c Department of Applied Chemistry, Cochin University of Science and Technology, Kochi 682 022, India

## Abstract

The title compound, C_15_H_13_N_3_O_3_, exists in the *E* conformation with respect to the azo­methane C=N double bond. The pyridyl and phenyl rings form dihedral angles of 35.67 (8) and 36.65 (7)°, respectively with the central C(=O)N_2_C unit. In the crystal, N—H⋯O and C—H⋯O hydrogen bonds connect the mol­ecules into chains along the *b* axis. Another C—H⋯O inter­action connects mol­ecules along the *c-*axis direction, forming layers.

## Related literature

For biological applications of benzohydrazones and their derivatives, see: Sreeja *et al.* (2004 ▶); Rakha *et al.* (1996 ▶); Takahama (1996 ▶). For the synthesis of related compounds, see: Emmanuel *et al.* (2011 ▶). For related structures, see: Reshma *et al.* (2012 ▶).
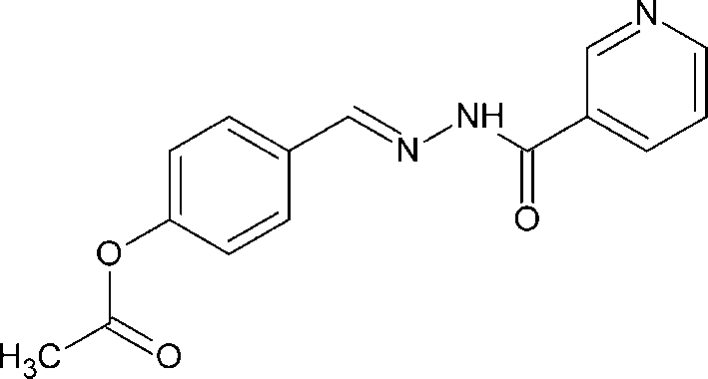


## Experimental

### 

#### Crystal data

C_15_H_13_N_3_O_3_
*M* *_r_* = 283.28
Monoclinic, 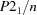

*a* = 16.347 (4) Å
*b* = 5.0859 (10) Å
*c* = 18.408 (5) Å
β = 115.311 (9)°
*V* = 1383.6 (6) Å^3^
*Z* = 4
Mo *K*α radiation
μ = 0.10 mm^−1^
*T* = 296 K
0.50 × 0.30 × 0.25 mm

#### Data collection

Bruker Kappa APEXII CCD diffractometer
Absorption correction: multi-scan (*SADABS*; Bruker, 2004 ▶) *T*_min_ = 0.953, *T*_max_ = 0.976
9585 measured reflections
3325 independent reflections
2288 reflections with *I* > 2σ(*I*)
*R*_int_ = 0.023

#### Refinement

*R*[*F* ^2^ > 2σ(*F* ^2^)] = 0.049
*wR*(*F* ^2^) = 0.140
*S* = 1.04
3325 reflections
195 parameters
1 restraint
H atoms treated by a mixture of independent and constrained refinement
Δρ_max_ = 0.31 e Å^−3^
Δρ_min_ = −0.25 e Å^−3^

### 

Data collection: *APEX2* (Bruker, 2004 ▶); cell refinement: *APEX2* and *SAINT* (Bruker, 2004 ▶); data reduction: *SAINT* and *XPREP* (Bruker, 2004 ▶); program(s) used to solve structure: *SHELXS97* (Sheldrick, 2008 ▶); program(s) used to refine structure: *SHELXL97* (Sheldrick, 2008 ▶); molecular graphics: *ORTEP-3* (Farrugia, 2012 ▶) and *DIAMOND* (Brandenburg, 2010 ▶); software used to prepare material for publication: *SHELXL97* and *publCIF* (Westrip, 2010 ▶).

## Supplementary Material

Crystal structure: contains datablock(s) Global, I. DOI: 10.1107/S1600536813025075/fj2641sup1.cif

Structure factors: contains datablock(s) I. DOI: 10.1107/S1600536813025075/fj2641Isup2.hkl

Supplementary material file. DOI: 10.1107/S1600536813025075/fj2641Isup3.cml

Additional supplementary materials:  crystallographic information; 3D view; checkCIF report

## Figures and Tables

**Table 1:** Hydrogen-bond geometry (Å, °)

*D*—H⋯*A*	*D*—H	H⋯*A*	*D*⋯*A*	*D*—H⋯*A*
N2—H2′⋯O1^i^	0.87 (1)	2.08 (1)	2.9107 (18)	162 (2)
C7—H7⋯O1^i^	0.93	2.48	3.251 (2)	140
C15—H15*C*⋯O3^ii^	0.96	2.55	3.469 (4)	161

Symmetry codes: (i) 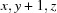; (ii) 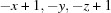.

**Fig. 1.**
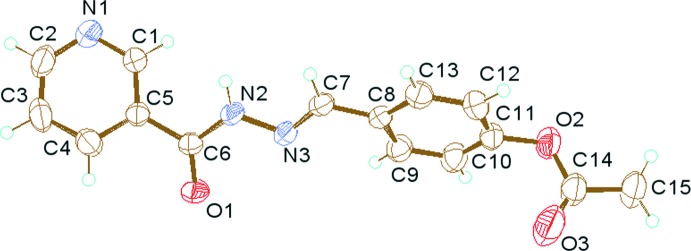
ORTEP view of the title compound drawn with 50% probability displacement ellipsoids for the non-H atoms.

**Fig. 2.**
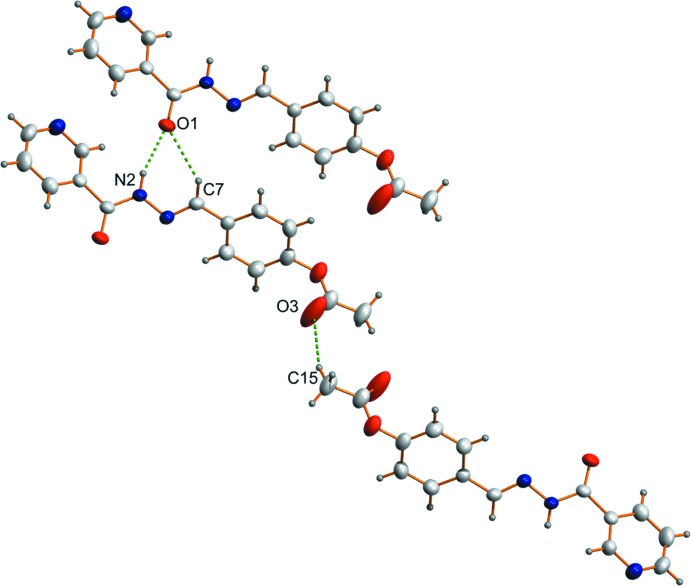
Hydrogen-bonding interactions in the crystal structure of C15H13N3O3.

**Fig. 3.**
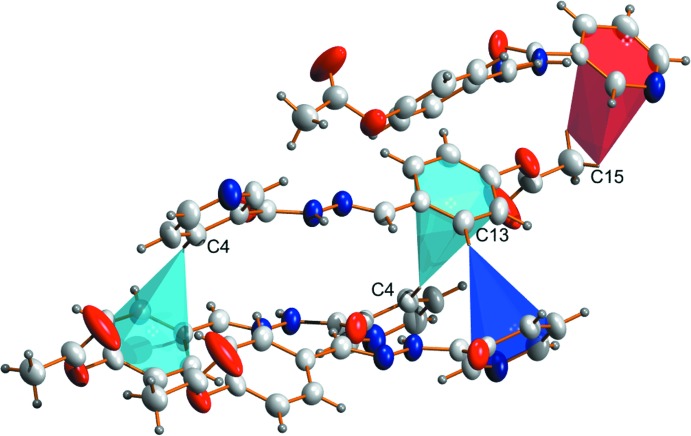
π···π interactions in the crystal structure of C15H13N3O3.

**Fig. 4.**
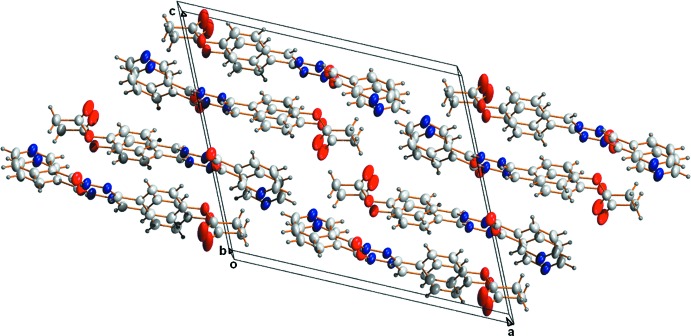
Packing diagram of the compound along the b axis.

## supplementary crystallographic information

### 1. Comment

Hydrazone derivatives show excellent spectrum of biological activities (Sreeja *et al.*, 2004). The chemical and pharmacological properties of aroylhydrazones have been extensively investigated recently owing to their potential application as antineoplastic, antiviral and antiinflammatory agents (Rakha *et al.*, 1996; Takahama, 1996).

The compound (Fig. 1) crystallizes in the monoclinic space group *P*2_1_/n. This molecule adopts an *E* configuration with respect to the C7=N3 bond and it exists in the amido form with a C6=O1 bond length of 1.2277 (18) Å which is very close to the reported C=O bond length of a related structure (Reshma *et al.*, 2012). The O1 and N2 atoms are in a *Z* configuration with respect to C6–N2 having a torsion angle of 9.9 (3)°. The central C(=O)N_2_C unit has dihedral angles of 35.67 (8) and 36.65 (7)°, respectively with the pyridyl and the phenyl rings.

There is a classical intermolecular N–H···O hydrogen bond interaction between the H atom attached at the N2 and O1 atom of neighbouring molecule with D···A distance of 2.9107 (18) Å and two C–H···O hydrogen bond interactions (Fig. 2) between the H atoms attached at the C7 & C15 and O1 & O3 atoms of neighbouring molecules with D···A distances of 3.251 (2) and 3.469 (4) Å, respectively. The classical hydrogen bond together with C–H···O interaction connect the molecules along *b* axis while the other C–H···O interaction chain the molecules along *c* axis in the crystal system. Four types of C–H···π interactions are also found in the molecular system (Fig. 3) with H···*Cg* distances of 3.1267, 3.2825, 3.3911 and 3.1984 Å forming a three-dimensional-supramolecuar architecture together with the intermolecular hydrogen bonding interactions. Although there are very weak short ring interactions found in the crystal system, they are not significant to support the network since centroid-centroid distances are above 4 Å. Fig. 4 shows a packing diagram of the title compound viewed along the *b* axis.

### 2. Experimental

The title compound was prepared by adapting a reported procedure (Emmanuel *et al.*, 2011). A solution of pyridine-3-carbohydrazide (0.137 g, 1 mmol) in ethanol (10 ml) was mixed with a methanolic solution (10 ml) of 4-formylphenyl acetate (0.164 g, 1 mmol). The mixture was refluxed for 6 h after adding few drops of glacial acetic acid and then cooled to room temperature. The formed crystals were collected, washed with few drops of methanol and dried over P_4_O_10_*in vacuo*. Colorless block shaped crystals, suitable for SXRD studies, were obtained after slow evaporation of the solution in air for a few days.

### 3. Refinement

The atom H2' was located from a difference Fourier map and N—H3' distance was restrained to 0.88±0.01 Å. The H atoms on C were placed in calculated positions, guided by difference maps, with C–H bond distances 0.93–0.96 Å. H atoms were assigned as *U*_iso_(H)=1.2Ueq(carrier) or 1.5Ueq (methyl C). Omitted owing to bad disagreement were the reflections (0 0 2) and (-1 0 1).

### Crystal data

**Table:**

C_15_H_13_N_3_O_3_	*F*(000) = 592
*M**_r_* = 283.28	*D*_x_ = 1.360 Mg m^−^^3^
Monoclinic, *P*2_1_/*n*	Mo *K*α radiation, λ = 0.71073 Å
Hall symbol: -P 2yn	Cell parameters from 2983 reflections
*a* = 16.347 (4) Å	θ = 2.8–26.9°
*b* = 5.0859 (10) Å	µ = 0.10 mm^−^^1^
*c* = 18.408 (5) Å	*T* = 296 K
β = 115.311 (9)°	Block, colorless
*V* = 1383.6 (6) Å^3^	0.50 × 0.30 × 0.25 mm
*Z* = 4	

### Data collection

**Table:**

Bruker Kappa APEXII CCD diffractometer	3325 independent reflections
Radiation source: fine-focus sealed tube	2288 reflections with *I* > 2σ(*I*)
Graphite monochromator	*R*_int_ = 0.023
Detector resolution: 8.33 pixels mm^-1^	θ_max_ = 28.0°, θ_min_ = 2.2°
ω and φ scan	*h* = −21→19
Absorption correction: multi-scan (*SADABS*; Bruker, 2004)	*k* = −6→6
*T*_min_ = 0.953, *T*_max_ = 0.976	*l* = −23→24
9585 measured reflections	

### Refinement

**Table:**

Refinement on *F*^2^	Primary atom site location: structure-invariant direct methods
Least-squares matrix: full	Secondary atom site location: difference Fourier map
*R*[*F*^2^ > 2σ(*F*^2^)] = 0.049	Hydrogen site location: inferred from neighbouring sites
*wR*(*F*^2^) = 0.140	H atoms treated by a mixture of independent and constrained refinement
*S* = 1.04	*w* = 1/[σ^2^(*F*_o_^2^) + (0.0632*P*)^2^ + 0.3098*P*] where *P* = (*F*_o_^2^ + 2*F*_c_^2^)/3
3325 reflections	(Δ/σ)_max_ < 0.001
195 parameters	Δρ_max_ = 0.31 e Å^−^^3^
1 restraint	Δρ_min_ = −0.25 e Å^−^^3^

### Special details

**Table:**

Geometry. All e.s.d.'s (except the e.s.d. in the dihedral angle between two l.s. planes) are estimated using the full covariance matrix. The cell e.s.d.'s are taken into account individually in the estimation of e.s.d.'s in distances, angles and torsion angles; correlations between e.s.d.'s in cell parameters are only used when they are defined by crystal symmetry. An approximate (isotropic) treatment of cell e.s.d.'s is used for estimating e.s.d.'s involving l.s. planes.
Refinement. Refinement of *F*^2^ against ALL reflections. The weighted *R*-factor *wR* and goodness of fit *S* are based on *F*^2^, conventional *R*-factors *R* are based on *F*, with *F* set to zero for negative *F*^2^. The threshold expression of *F*^2^ > σ(*F*^2^) is used only for calculating *R*-factors(gt) *etc*. and is not relevant to the choice of reflections for refinement. *R*-factors based on *F*^2^ are statistically about twice as large as those based on *F*, and *R*- factors based on ALL data will be even larger.

### Fractional atomic coordinates and isotropic or equivalent isotropic displacement parameters (Å^2^)

**Table:**

	*x*	*y*	*z*	*U*_iso_*/*U*_eq_	
O1	0.62319 (9)	−0.2235 (2)	−0.00860 (7)	0.0554 (4)	
O2	0.64307 (10)	0.4099 (3)	0.44141 (7)	0.0639 (4)	
N1	0.72016 (12)	0.3431 (3)	−0.15907 (10)	0.0597 (4)	
N2	0.62677 (10)	0.2123 (3)	0.01908 (8)	0.0427 (3)	
N3	0.62691 (10)	0.1739 (3)	0.09359 (8)	0.0420 (3)	
C1	0.70076 (13)	0.2839 (3)	−0.09748 (10)	0.0477 (4)	
H1	0.7253	0.3894	−0.0519	0.057*	
C2	0.68290 (15)	0.1905 (4)	−0.22385 (11)	0.0603 (5)	
H2	0.6949	0.2292	−0.2677	0.072*	
C3	0.62814 (14)	−0.0194 (4)	−0.22982 (11)	0.0596 (5)	
H3	0.6035	−0.1191	−0.2766	0.071*	
C4	0.61008 (12)	−0.0806 (4)	−0.16510 (10)	0.0493 (4)	
H4	0.5741	−0.2244	−0.1671	0.059*	
C5	0.64659 (11)	0.0761 (3)	−0.09706 (9)	0.0365 (4)	
C6	0.63100 (11)	0.0061 (3)	−0.02519 (10)	0.0380 (4)	
C7	0.60960 (11)	0.3795 (3)	0.12422 (10)	0.0403 (4)	
H7	0.5928	0.5334	0.0942	0.048*	
C8	0.61583 (10)	0.3768 (3)	0.20592 (9)	0.0373 (4)	
C9	0.66527 (12)	0.1856 (3)	0.26163 (10)	0.0457 (4)	
H9	0.6934	0.0516	0.2464	0.055*	
C10	0.67294 (12)	0.1932 (4)	0.33914 (10)	0.0510 (4)	
H10	0.7064	0.0660	0.3762	0.061*	
C11	0.63065 (12)	0.3906 (4)	0.36095 (10)	0.0477 (4)	
C12	0.58098 (13)	0.5817 (4)	0.30759 (12)	0.0537 (5)	
H12	0.5522	0.7130	0.3232	0.064*	
C13	0.57459 (13)	0.5752 (3)	0.23019 (11)	0.0485 (4)	
H13	0.5422	0.7056	0.1939	0.058*	
C14	0.59039 (13)	0.2668 (4)	0.46456 (11)	0.0568 (5)	
C15	0.61036 (14)	0.3114 (5)	0.55032 (11)	0.0647 (6)	
H15A	0.6115	0.4968	0.5605	0.097*	
H15B	0.6681	0.2362	0.5840	0.097*	
H15C	0.5643	0.2299	0.5619	0.097*	
H2'	0.6234 (12)	0.369 (2)	−0.0002 (10)	0.048 (5)*	
O3	0.53569 (15)	0.1229 (5)	0.41928 (11)	0.1332 (10)	

### Atomic displacement parameters (Å^2^)

**Table:**

	*U*^11^	*U*^22^	*U*^33^	*U*^12^	*U*^13^	*U*^23^
O1	0.0910 (10)	0.0271 (6)	0.0590 (8)	−0.0011 (6)	0.0423 (7)	0.0038 (5)
O2	0.0756 (9)	0.0812 (10)	0.0411 (7)	−0.0283 (8)	0.0308 (7)	−0.0160 (7)
N1	0.0870 (12)	0.0523 (9)	0.0551 (9)	−0.0080 (8)	0.0450 (9)	0.0004 (8)
N2	0.0680 (9)	0.0283 (7)	0.0422 (8)	0.0018 (6)	0.0335 (7)	0.0051 (6)
N3	0.0589 (9)	0.0354 (7)	0.0401 (7)	−0.0009 (6)	0.0292 (6)	0.0034 (6)
C1	0.0674 (11)	0.0391 (9)	0.0435 (9)	−0.0065 (8)	0.0304 (9)	−0.0046 (7)
C2	0.0835 (14)	0.0644 (12)	0.0435 (10)	0.0063 (11)	0.0373 (10)	0.0064 (9)
C3	0.0724 (13)	0.0663 (13)	0.0356 (9)	0.0018 (10)	0.0189 (9)	−0.0102 (9)
C4	0.0540 (10)	0.0445 (9)	0.0458 (10)	−0.0027 (8)	0.0180 (8)	−0.0067 (8)
C5	0.0452 (8)	0.0296 (7)	0.0353 (8)	0.0060 (6)	0.0178 (7)	0.0034 (6)
C6	0.0484 (9)	0.0280 (8)	0.0399 (8)	0.0014 (6)	0.0209 (7)	0.0037 (6)
C7	0.0502 (9)	0.0336 (8)	0.0426 (9)	−0.0010 (7)	0.0252 (7)	0.0053 (7)
C8	0.0429 (8)	0.0349 (8)	0.0403 (8)	−0.0055 (6)	0.0237 (7)	−0.0007 (6)
C9	0.0494 (9)	0.0449 (9)	0.0471 (10)	0.0039 (7)	0.0248 (8)	0.0017 (8)
C10	0.0560 (10)	0.0550 (11)	0.0404 (9)	0.0027 (8)	0.0190 (8)	0.0061 (8)
C11	0.0543 (10)	0.0549 (11)	0.0384 (9)	−0.0167 (8)	0.0240 (8)	−0.0100 (8)
C12	0.0691 (12)	0.0469 (10)	0.0570 (11)	−0.0012 (9)	0.0384 (10)	−0.0091 (9)
C13	0.0602 (11)	0.0388 (9)	0.0513 (10)	0.0034 (8)	0.0285 (8)	0.0018 (8)
C14	0.0563 (11)	0.0758 (13)	0.0406 (9)	−0.0116 (10)	0.0230 (8)	−0.0064 (9)
C15	0.0679 (13)	0.0904 (16)	0.0411 (10)	−0.0038 (11)	0.0282 (9)	−0.0026 (10)
O3	0.1493 (18)	0.203 (2)	0.0656 (11)	−0.1225 (18)	0.0636 (12)	−0.0513 (13)

### Geometric parameters (Å, º)

**Table:**

O1—C6	1.2277 (18)	C7—C8	1.463 (2)
O2—C14	1.329 (2)	C7—H7	0.9300
O2—C11	1.410 (2)	C8—C13	1.389 (2)
N1—C2	1.332 (3)	C8—C9	1.394 (2)
N1—C1	1.336 (2)	C9—C10	1.378 (2)
N2—C6	1.348 (2)	C9—H9	0.9300
N2—N3	1.3844 (19)	C10—C11	1.373 (3)
N2—H2'	0.866 (9)	C10—H10	0.9300
N3—C7	1.276 (2)	C11—C12	1.374 (3)
C1—C5	1.381 (2)	C12—C13	1.384 (3)
C1—H1	0.9300	C12—H12	0.9300
C2—C3	1.367 (3)	C13—H13	0.9300
C2—H2	0.9300	C14—O3	1.180 (2)
C3—C4	1.380 (3)	C14—C15	1.486 (3)
C3—H3	0.9300	C15—H15A	0.9600
C4—C5	1.386 (2)	C15—H15B	0.9600
C4—H4	0.9300	C15—H15C	0.9600
C5—C6	1.493 (2)		
			
C14—O2—C11	118.47 (14)	C13—C8—C9	118.60 (16)
C2—N1—C1	116.41 (17)	C13—C8—C7	119.60 (15)
C6—N2—N3	120.70 (13)	C9—C8—C7	121.75 (15)
C6—N2—H2'	118.8 (12)	C10—C9—C8	120.64 (16)
N3—N2—H2'	120.5 (12)	C10—C9—H9	119.7
C7—N3—N2	114.55 (13)	C8—C9—H9	119.7
N1—C1—C5	124.26 (16)	C11—C10—C9	119.27 (17)
N1—C1—H1	117.9	C11—C10—H10	120.4
C5—C1—H1	117.9	C9—C10—H10	120.4
N1—C2—C3	124.06 (18)	C10—C11—C12	121.74 (16)
N1—C2—H2	118.0	C10—C11—O2	119.60 (17)
C3—C2—H2	118.0	C12—C11—O2	118.53 (17)
C2—C3—C4	118.76 (17)	C11—C12—C13	118.72 (17)
C2—C3—H3	120.6	C11—C12—H12	120.6
C4—C3—H3	120.6	C13—C12—H12	120.6
C3—C4—C5	118.82 (18)	C12—C13—C8	121.02 (17)
C3—C4—H4	120.6	C12—C13—H13	119.5
C5—C4—H4	120.6	C8—C13—H13	119.5
C1—C5—C4	117.67 (16)	O3—C14—O2	120.82 (18)
C1—C5—C6	122.69 (14)	O3—C14—C15	126.92 (19)
C4—C5—C6	119.53 (15)	O2—C14—C15	112.26 (17)
O1—C6—N2	123.50 (15)	C14—C15—H15A	109.5
O1—C6—C5	121.50 (14)	C14—C15—H15B	109.5
N2—C6—C5	115.00 (13)	H15A—C15—H15B	109.5
N3—C7—C8	121.05 (14)	C14—C15—H15C	109.5
N3—C7—H7	119.5	H15A—C15—H15C	109.5
C8—C7—H7	119.5	H15B—C15—H15C	109.5
			
C6—N2—N3—C7	−170.37 (15)	N3—C7—C8—C13	−163.02 (16)
C2—N1—C1—C5	0.9 (3)	N3—C7—C8—C9	19.6 (2)
C1—N1—C2—C3	−0.7 (3)	C13—C8—C9—C10	0.1 (2)
N1—C2—C3—C4	−0.4 (3)	C7—C8—C9—C10	177.51 (15)
C2—C3—C4—C5	1.3 (3)	C8—C9—C10—C11	0.5 (3)
N1—C1—C5—C4	0.0 (3)	C9—C10—C11—C12	−0.2 (3)
N1—C1—C5—C6	176.14 (16)	C9—C10—C11—O2	−175.82 (15)
C3—C4—C5—C1	−1.1 (2)	C14—O2—C11—C10	−85.1 (2)
C3—C4—C5—C6	−177.41 (16)	C14—O2—C11—C12	99.2 (2)
N3—N2—C6—O1	9.9 (3)	C10—C11—C12—C13	−0.7 (3)
N3—N2—C6—C5	−169.77 (13)	O2—C11—C12—C13	174.95 (16)
C1—C5—C6—O1	−144.25 (18)	C11—C12—C13—C8	1.3 (3)
C4—C5—C6—O1	31.8 (2)	C9—C8—C13—C12	−1.0 (3)
C1—C5—C6—N2	35.5 (2)	C7—C8—C13—C12	−178.50 (16)
C4—C5—C6—N2	−148.44 (16)	C11—O2—C14—O3	1.5 (3)
N2—N3—C7—C8	−174.03 (14)	C11—O2—C14—C15	−179.02 (17)

### Hydrogen-bond geometry (Å, º)

Cg1and Cg2 are the centroids of the C8–C13 and C1–C5/N1 rings, respectively.

**Table:**

*D*—H···*A*	*D*—H	H···*A*	*D*···*A*	*D*—H···*A*
N2—H2′···O1^i^	0.87 (1)	2.08 (1)	2.9107 (18)	162 (2)
C7—H7···O1^i^	0.93	2.48	3.251 (2)	140
C15—H15*C*···O3^ii^	0.96	2.55	3.469 (4)	161
C4—H4···*Cg*1^iii^	0.93	3.13	3.821 (2)	133
C13—H13···*Cg*2^iii^	0.93	3.28	3.860 (3)	122
C15—H15*A*···*Cg*2^iv^	0.96	3.39	3.734 (3)	104
C15—H15*B*···*Cg*2^iv^	0.96	3.20	3.734 (3)	117

Symmetry codes: (i) *x*, *y*+1, *z*; (ii) −*x*+1, −*y*, −*z*+1; (iii) −*x*+1, −*y*, −*z*; (iv) −*x*+3/2, *y*+1/2, −*z*+1/2.
